# Vestibular Symptoms After Cochlear Implantation in Patients With Meniere Disease

**DOI:** 10.1097/ONO.0000000000000074

**Published:** 2025-08-27

**Authors:** Alexandra M. Arambula, Claudia I. Cabrera, John E. Richter, Sarah E. Mowry

**Affiliations:** 1 Department of Otolaryngology-Head and Neck Surgery, University Hospitals Cleveland Medical Center, Cleveland, Ohio; 2 Department of Otolaryngology-Head and Neck Surgery, Case Western Reserve University School of Medicine, Cleveland, Ohio.

**Keywords:** Cochlear implant, Meniere disease, Migraine, Vestibular dysfunction

## Abstract

**Objective:**

To evaluate the impact of cochlear implantation (CI) on vestibular symptoms in patients with Meniere disease (MD).

**Study Design:**

Retrospective chart review.

**Setting:**

Single tertiary referral center.

**Patients:**

Patients with MD, who underwent CI between 2011 and 2024 and had documentation of preoperative and postoperative vestibular symptoms.

**Intervention:**

Placement of a cochlear implant for hearing rehabilitation.

**Main Outcome Measure:**

Preoperative and postoperative subjective vestibular symptom severity.

**Results:**

Of 39 patients meeting inclusion criteria, 51% had moderate or severe vestibular symptoms preoperatively and 41% had mild symptoms. After CI, 28.2% had symptom improvement, 64.1% had stable symptoms, and 7.7% had symptom worsening. If preoperative symptom severity was mild or absent, compared with moderate or severe, patients were more likely to have stable symptoms (78.9% versus 50.0%, respectively) and less likely to experience symptom improvement (10.5% versus 45.0%, respectively), *P* = 0.038. Significantly more patients with moderate or severe preoperative symptoms had migraines compared with those with mild or absent symptoms (35.0% versus 5.3%, respectively, *P* = 0.044). Among patients with moderate or severe symptoms, those without comorbid migraines were more likely to experience symptom improvement compared with those with migraines (*P* = 0.0031).

**Conclusions:**

Over 90% of MD patients who underwent CI in the MD-affected ear experienced vestibular symptom stability or improvement postoperatively. Patients with more severe preoperative symptoms were more likely to experience symptom improvement if they did not have comorbid migraines. These findings may have important implications for preoperative patient counseling and require validation in a larger patient cohort with objective vestibular symptom metrics.

Meniere disease (MD), a condition of the inner ear characterized by endolymphatic hydrops, presents with episodic vertigo, hearing loss, tinnitus, and aural pressure (1). Establishing a definite MD diagnosis requires 2 or more documented episodes of vertigo lasting from 20 minutes to 12 hours, audiometrically recorded low- to medium-frequency sensorineural hearing loss in the affected ear, and the presence of fluctuating aural symptoms, like tinnitus and aural pressure. Additionally, the symptoms must not be better attributed to another vestibular diagnosis, such as benign paroxysmal positional vertigo or vestibular schwannoma (2).

In patients with MD, hearing loss classically occurs in a relapsing-remitting fashion, worsening during crises and improving afterward. Hearing loss is typically progressive over time, and affected patients benefit from traditional amplification or eventual placement of a cochlear implant (CI) (1). Use of a CI may improve speech recognition, tinnitus, and quality of life for qualified MD patients (3,4). Hearing benefits appear comparable to those seen in controls without MD, even if labyrinthectomy has also been performed (3).

Surgery to place a CI may be performed alongside related MD procedures, such as labyrinthectomy, endolymphatic sac (ELS) decompression, or vestibular neurectomy (5,6). These latter procedures are indicated for relief of MD vestibular symptoms. However, some studies have suggested that cochlear implantation alone can also reduce vestibular symptom severity (3,7). The objective of the present study is to describe our institution’s experience with vestibular symptomatology following cochlear implantation in MD patients. Based on clinical experience, we hypothesized that cochlear implantation would improve vestibular symptoms in MD patients.

## MATERIALS AND METHODS

This study was approved by the University Hospitals Cleveland Medical Center Institutional Review Board under IRB STUDY20240770.

A retrospective review of patient records from 2011 to 2024 was performed at a single, tertiary referral center. Patients were selected from an existing prospective database of patients who underwent cochlear implantation at our institution. This initial list was supplemented with an additional search of the electronic medical record, performed using the Electronic Medical Record Search Engine research tool (8), with search terms of (“meniere’s” [and other synonyms provided by the program]) AND (“cochlear implant”). Patients were included if they were 18 years or older, underwent cochlear implantation in the MD-affected ear, and had a diagnosis of definite or probable MD (as defined by the American Academy of Otolaryngology—Head and Neck Surgery Clinical Practice Guideline) (9). Patients were excluded if their diagnosis was not MD (eg, cochlear hydrops, Meniere syndrome), if their CI was in the non-MD ear, if the CI was performed at an outside institution precluding assessment of preoperative symptomatology, and if their subjective preoperative and/or postoperative data were not available for review. We collected demographic information, neurologic comorbidities, previous surgical interventions for MD, and preoperative and 6-month postoperative AzBio scores (in quiet) and pure tone averages (PTA). PTA was calculated as the average of pure tone thresholds at 500, 1000, and 2000 Hz. Comorbid neurological conditions included vestibular migraine (VM), history of traumatic brain injury, cognitive impairment, seizure disorder or epilepsy, prior cerebrovascular accident (CVA) or transient ischemic attack, brain mass, and prior intracranial surgery or radiation.

Patient charts were reviewed for reports of vestibular symptoms and the impact of these symptoms on their daily lives. Vestibular symptoms were categorized by 2 authors (A.M.A. and S.E.M.) as “severe,” “moderate,” “mild,” or “none” both before and after cochlear implantation. “Severe” disease included frequent vertigo spells that significantly impacted activities of daily living several days a week and/or resulted in harm events (ie, falls). “Moderate” disease included vertigo spells or disequilibrium with or without vertigo that did not significantly interfere with activities of daily living or that affected patients up to once a week. “Mild” disease included infrequent vertigo (less than twice per month), as well as long periods of disease quiescence. “None” was defined as no active vestibular symptoms. Charts were also surveyed for preoperative and postoperative Dizziness Handicap Index (DHI) scores when available.

Patients’ vestibular symptoms were compared pre- and postoperatively to categorize patients based on whether their symptoms improved, worsened, or did not change. Improvement or worsening required a change in the symptom nominal category (severe, moderate, mild, or none) after cochlear implantation. Associations between preoperative and postoperative vestibular symptom severity, presence of migraine or other neurologic comorbidity, and postoperative vestibular symptom status were explored. Due to very small sample sizes observed across categorical variables in this study (ie, frequent n = 0 and n < 5), the “none” and “mild” categories were collapsed together, and the “moderate” and “severe” categories were collapsed together before performing our comparative statistical analyses. For categorical data, the Yates chi-squared test and Fisher exact test were used for parametric and nonparametric data, respectively. For any category with a sample size n < 5, Fisher exact test was used. For continuous data, the Student *t* test and Kruskal–Wallis test were used for parametric and nonparametric data, respectively. Calculations were performed with R Statistical Software (v4.2.1; R Core Team 2022). Statistical significance was defined as *P* < 0.05.

## RESULTS

During the specified study period, 98 patients were identified, of which 59 were excluded due to missing vestibular data, lack of definite or probable MD diagnosis, or CI placement in the non-MD ear. Table 1 details demographic and preoperative data of the 39 patients included for analysis. There was a nearly even distribution of males and females. MD was bilateral in approximately 25% (10/39) of cases, resulting in 49 MD-affected ears in this cohort. The majority of patients (23/39, 59%) had asymmetric sensorineural hearing loss and underwent unilateral cochlear implantation (35/39, 89.7%) at a median age of 68 years (interquartile range [IQR]: 62–75 years). DHI data were excluded, as it was only available at one time point for 9 patients. Review of subjective preoperative vestibular data revealed that 51% of patients had moderate or severe symptoms, 41% had mild symptomatology, and 9% had no active vestibular symptoms. One-third of patients underwent ELS decompression with/without shunt placement and/or labyrinthectomy for their MD. Three patients had a labyrinthectomy and 3 had an ELS decompression performed at the time of their CI; the remainder had their ELS decompression or labyrinthectomy performed before their CI. Twenty percent of the group (8/39) had comorbid migraines. Two of these patients with migraines also had superior semicircular canal dehiscence. An additional 5 patients had at least one other neurologic comorbidity, including contralateral vestibular schwannoma (2 patients), cognitive dysfunction, seizures, CVA, myasthenia gravis, and Charcot–Marie–Tooth disorder.

**TABLE 1.** Demographics and preoperative and postoperative audiometric data of 39 patients with MD who underwent CI in the MD-affected ear

		MD preoperative vestibular symptom severity				
	All patients (N = 39)	None (n = 3)	Mild (n = 16)	Moderate (n = 10)	Severe (n = 10)	*P* value
Age at time of CI, y; median (IQR)	68 (62–75)	70 (66–73)	72 (66–77)	65 (59–69)	64 (54–71)	0.14
Gender, n (%)						0.41
Female	19 (48.7)	1 (33.3)	6 (37.5)	5 (50.0)	7 (70.0)	
Male	20 (51.3)	2 (66.7)	10 (62.5)	5 (50.0)	3 (30.0)	
What type of hearing loss does this patient have, n (%)						0.67
Asymmetric sensorineural hearing loss	23 (59.0)	2 (66.7)	10 (62.5)	4 (40.0)	7 (70.0)	
Bilateral sensorineural hearing loss	14 (35.9)	1 (33.3)	6 (37.5)	5 (50.0)	2 (20.0)	
Unilateral sensorineural hearing loss (single-sided deafness)	2 (5.1)	0 (0.0)	0 (0.0)	3 (30.0)	1 (10.0)	
Tinnitus, n (%)						0.50
Yes	31 (83.8)	2 (66.7)	12 (75.0)	9 (90.0)	9 (90.0)	
No	6 (16.2)	1 (33.3)	4 (25.0)	1 (10.0)	1 (10.0)	
CI received, n (%)						0.066
Unilateral	35 (89.7)	3 (100.0)	16 (100.0)	7 (70.0)	9 (90.0)	
Bilateral	4 (10.3)	0 (0.0)	0 (0.0)	3 (30.0)	1 (10.0)	
MD ear, n (%)						0.84
Right	10 (25.6)	1 (33.3)	5 (31.2)	1 (10.0)	3 (30.0)	
Left	19 (48.7)	1 (33.3)	8 (50.0)	5 (50.0)	5 (50.0)	
Bilateral	10 (25.6)	1 (33.3)	3 (18.8)	4 (40.0)	2 (20.0)	
Neurologic comorbidity, n (%)	6 (15.4)	0 (0.0)	4 (25.0)	1 (10.0)	1 (10.0)	0.77
Migraine comorbidity, n (%)	8 (20.5)	0 (0.0)	1 (6.3)	2 (20.0)	5 (50.0)	**0.049**
Preoperative AzBio +10 SNR, % correct; median (IQR)		3 (1.5–29.5)	2 (0.0–11.0)	6 (0.0– 12.0)	8 (0.0– 66.5)	0.82
Postoperative AzBio +10 SNR, % correct; median (IQR)		66 (60.5–72.5)	48 (34.3–62.3)	72 (63.5–90.0)	87 (35.5–93.5)	0.11
Preoperative PTA, dB HL; median (IQR)		69 (67.1–71.3)	84 (76.3–90.4)	72 (64.2–90.8)	78 (64.2–86.3)	0.30
Postoperative PTA, dB HL; median (IQR)		33 (30.8–34.2)	30 (25.8–31.7)	28 (26.7–30.0)	31 (28.8–32.9)	0.46
MD postoperative vestibular symptom status, n (%)						**0.038**
Improve	11 (28.2)	0 (0.0)	2 (12.5)	5 (50.0)	4 (40.0)	
Stable	25 (64.1)	3 (100.0)	12 (75.0)	4 (40.0)	6 (60.0)	
Worsen	3 (7.7)	0 (0.0)	2 (12.5)	1 (10.0)	0 (0.0)	

The data are also stratified by preoperative vestibular disease severity (none, mild, moderate, and severe). Four patients with bilateral MD underwent bilateral CI (totaling 43 ears in 39 patients); age at CI and preoperative and postoperative audiometric data points reflect the data for all ears. The remainder of the data fields are for n as specified in the column headings. Statistical significance was defined as *P* < 0.05 (indicated in bold).

CI indicates cochlear implantation; dB HL, decibels hearing level; IQR, interquartile range; MD, Meniere disease; PTA, pure tone average; Q, quiet; SNR, signal-to-noise ratio.

When comparing patients based on preoperative disease severity, there was no significant difference in gender distribution, MD laterality, presence of neurologic comorbidity, or preoperative PTA or AzBio scores (Table 1). Patients with moderate and severe preoperative symptoms were significantly younger than those with mild or absent symptoms (median: 65 versus 70 years, *P* = 0.020). Significantly more patients with moderate and severe symptoms had comorbid migraines than did those with mild or absent symptoms (35.0% versus 5.3%, *P* = 0.044). Postoperative PTA scores were not significantly different between patients based on preoperative symptom severity (Table 1). However, patients with moderate or severe preoperative symptoms did have significantly worse AzBio scores postoperatively (median: 58% AzBio in quiet [37.0%–77.5%]), compared with patients with mild or absent preoperative symptoms (median: 86% AzBio in quiet [75.5%–90.5%]), *P* = 0.034.

**FIG. 1.**
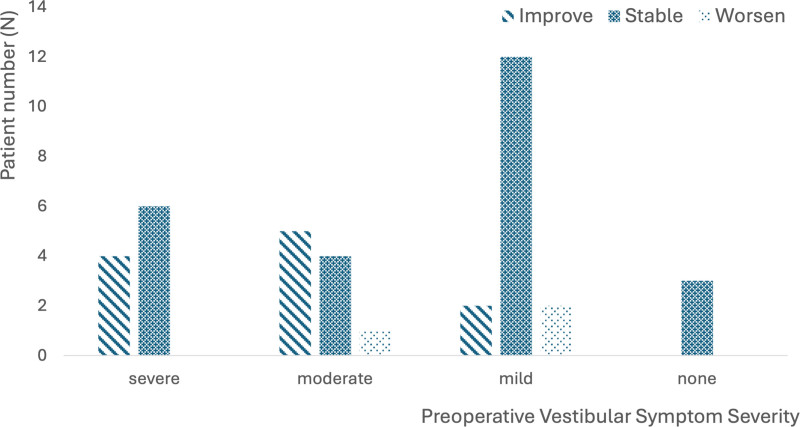
Vestibular symptom status (improvement, stability, or worsening) after cochlear implantation, stratified by preoperative symptom severity. There was a statistically significant difference in vestibular outcomes when comparing those with severe and moderate symptoms to those with mild or absent symptoms (*P* = 0.038): those with mild or absent preoperative symptoms were more likely to have stable vestibular symptoms after cochlear implantation, while those with moderate or severe symptoms were more likely to experience improvement in their symptoms.

In the entire patient cohort, 11/39 (28.2%) experienced improvement in vestibular symptoms, 25/39 (64.1%) experienced no change, and the remaining 3 patients had worsening of their symptoms. When stratified based on preoperative symptom severity, all patients without preoperative symptoms remained stable. Most patients with mild symptoms remained stable postoperatively (12/16, 75.0%), 2 patients improved from mild to no symptoms, and 2 patients worsened from mild to moderate symptom severity. One of the 2 patients with worsening of symptoms had a contralateral vestibular schwannoma that was previously excised and a cerebellar infarction approximately 4 months postoperatively; thus, it is difficult to fully attribute their change in symptoms to the MD and CI. Among those with moderate or severe vestibular symptoms, every patient experienced stability (10/20, 50.0%) or improvement (9/20, 45.0%), except for 1 patient who declined from moderate to severe symptom severity. The patient who had worsened symptoms had bilateral MD and received a unilateral CI, with resultant bilateral vestibular hypofunction. Postoperative symptoms were significantly associated with the preoperative symptom stratification; those with mild/no symptoms preoperatively were more likely to have stable vestibular symptoms, whereas those with moderate/severe disease were more likely to experience improvement (*P* = 0.38, Table 1 and Fig. 1).

**FIG. 2.**
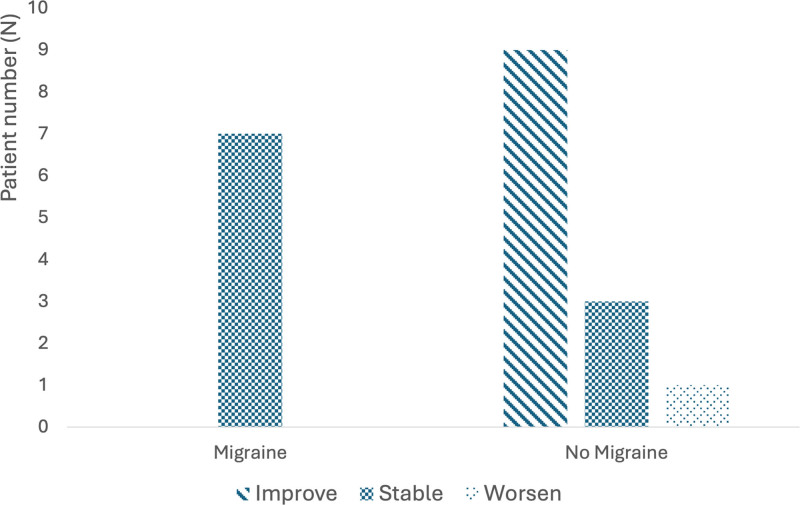
Vestibular symptom status (improvement, stability, or worsening) after cochlear implantation in patients with moderate or severe preoperative symptoms, stratified by the presence or absence of comorbid migraines. There was a statistically significant difference in vestibular outcomes for those with and without migraines (*P* = 0.0031): those with migraines were more likely to have persistent moderate/severe symptoms after cochlear implantation, while those without migraines were more likely to experience symptom improvement.

Because there was a significant difference in migraine prevalence based on preoperative symptom severity, we assessed the change in symptoms for the severe and moderate groups stratified by the presence or absence of comorbid migraines. Seven patients with severe or moderate preoperative symptom severity had migraines and had persistent moderate or severe vestibular symptoms postoperatively. Of the 13 patients with severe or moderate preoperative symptoms without comorbid migraines, 9 (69.2%) had symptom improvement, 3 (23.1%) had no change in symptoms, and 1 (7.7%) had worsened symptoms (Fig. 2). This difference in postoperative outcome between patients with and without comorbid migraines was statistically significant (*P* = 0.0031). We did not perform a subgroup analysis for patients with mild or absent vestibular symptoms because only 1 patient in the mild group had comorbid migraines.

## DISCUSSION

In the present study, approximately 90% of patients with MD who underwent cochlear implantation in the MD-affected ear experienced stability or improvement in their vestibular symptoms. If preoperative vestibular symptom severity was mild or absent compared with moderate or severe, a higher percentage of patients had stable symptoms (78.9% versus 50.0%, respectively) and a lower percentage experienced symptom improvement (10.5% versus 45.0%, respectively). This observation is explained by a “ceiling effect” in patients with mild or absent preoperative vestibular symptoms. As those patients already have minimal vestibular symptoms, they may be less likely to experience clinically detectable or meaningful symptomatic improvement. Our study did also identify a significantly higher prevalence of comorbid migraines in patients with moderate or severe preoperative vestibular symptoms (35.0% versus 5.3% in patients with mild or absent preoperative symptoms). Among these patients with moderate or severe symptoms, those with comorbid migraines were significantly more likely to have persistent moderate/severe symptoms (100%) compared with those without migraines, who were more likely to experience symptom improvement (69.2%). There was a low rate of symptom worsening in the entire cohort (7.7%).

Several studies have commented on the beneficial CI-related hearing outcomes in patients with MD, which are similar to patients without MD (10–14). Our audiometric outcomes are similar to those published studies. Though not the focus of our study, we did note that patients with moderate or severe preoperative symptoms had significantly worse CI performance, based on AzBio scores, than did patients with mild or absent preoperative symptomatology. It is possible that our patients with moderate or severe symptoms were experiencing not only substantial damage to their vestibular system but also to their cochlear function and cochlear reserve. It is also possible that the increased cognitive load required to compensate for their more severe disequilibrium detracted from the mental energy that they could apply to their post-CI rehabilitation.

While hearing rehabilitation is an important component of the care of patients with MD, vestibular symptoms can be disabling and should be considered when selecting more invasive treatment options for this patient population, particularly because cochlear implantation may cause at least transient vestibular dysfunction in 32% to 74% of patients without MD who undergo CI (15–19). Relatively few studies exist describing the effects of CI on vestibular symptoms in MD-affected patients (10,11,13,14,20,21), and these published studies include fewer than 30 patients. Thus, our study’s findings are significant, as they add to the body of literature describing the effect of CI on vestibular symptomatology in patients with MD, and ours is one of the largest cohorts of this kind to date.

Comparison of our vestibular data to existing literature is somewhat limited, as the rating of vestibular impairment and the type of surgical intervention are inconsistent across studies. McRackan and colleagues (13) evaluated 21 patients with MD who underwent CI in the MD-affected ear. Only 6 (28.6%) of their patients had active MD symptoms and 83.3% of these 6 patients had resolution or improvement of their vestibular symptoms. Mukherjee and colleagues (10) evaluated 27 patients with MD who underwent CI with or without labyrinthectomy. In those who had a labyrinthectomy, there was no reported postoperative vestibular impairment. In 22 patients who underwent CI without labyrinthectomy, 55% experienced vestibular dysfunction postoperatively, with 6/22 (27.3%) of these patients experiencing more prolonged or delayed symptoms. Mick and colleagues (21) reported on a similar sample size of patients (20) with MD who underwent CI alone; 30% had preoperative dizziness and 20% had chronic postoperative dizziness. More recently, Yilmaz Topçuoğlu and colleagues (11) evaluated 11 MD-implanted ears and found significant improvement in vertigo impairment across the entire cohort, based on subjective visual analog scale scores (median of 9 preoperatively to 3 postoperatively) and DHI scores (median 65 points preoperatively to 37 points postoperatively). A systematic review from 2021 (14) found that of a pooled 182 patients with MD who underwent CI with or without labyrinthectomy, 69 patients (37.9%) had preoperative vertigo or severe dizziness and 22/182 (12.1%) had vertigo postoperatively. All patients with postoperative vertigo did not undergo labyrinthectomy, amounting to a slightly higher percentage (15.4%) of postoperative vertigo in patients who underwent CI without labyrinthectomy (22/143). Half of these 22 patients, or nearly 8% of the total cohort (11/143), experienced chronic vertigo.

Compared with the reported 8%–27.3% incidence of prolonged vestibular dysfunction after CI in MD patients (10,11,13,14), we had a slightly higher percentage of patients (35.9%) with chronic moderate or severe vestibular symptoms postoperatively. Only 28.6% of patients in McRackan’s study and 30% in Mick’s study had vestibular symptoms preoperatively, which is close to the pooled percentage of patients (37.9%) having preoperative vestibular symptoms in Desiato and colleagues’ systematic review (13,14,21). In contrast, 87.2% of our cohort had at least some preoperative disease activity, with 51.3% of our cohort having moderate or severe symptoms. Thus, our higher percentage of moderate or severe postoperative symptoms may be due, in part, to a patient population with more severe MD phenotypes compared with the other referenced studies. Additionally, these studies, with the exception of Yilmaz Topçuoğlu et al, do not specify the severity of pre- or postoperative vestibular symptoms (10–14,20,21), making comparison of our patient cohort to existing literature less straightforward.

Other existing literature does not comment on neurologic comorbidities in their MD patients, but our data documents a 20% prevalence of migraines in our cohort and a 33% prevalence of migraine and/or other neurologic comorbidities that could affect vestibular function. Migraine prevalence is approximately twice as high in patients with MD compared with the general population (22). With the increasing recognition of VM as a diagnostic entity, its symptomatic overlap with MD leads to confusion in its differentiation from MD or in diagnosis as a comorbidity with MD (23). Neff and colleagues (24) reported on 147 consecutive patients with MD, VM, or MD with VM and found that 25% of these patients had MD and VM. Because VM may contribute to chronic vestibular dysfunction, its consideration in the management of patients with MD is increasingly important. In our patients with moderate and severe vestibular symptoms, a comorbid migraine diagnosis was significantly associated with nonimprovement of vestibular symptoms after CI, compared with a higher likelihood of symptomatic improvement in patients without underlying migraines. Though this finding requires validation in a larger population, this may carry significant implications for patient counseling regarding anticipated postoperative vestibular symptoms.

This study has several limitations. Because of its retrospective nature, the available data for review were limited. We did not have adequate DHI or quality of life data available to include in this study, and we had to rely on subjective interpretation of patient charts to obtain information on vestibular symptomatology. This certainly introduces potential bias from the authors reviewing patient charts. While objective vestibular testing data would strengthen this study, we also highlight that subjective patient data is important for consideration, as we ultimately must consider a patient’s lived experience in their management, particularly if that information does not align with an objective metric. Future analyses should incorporate not only this subjective data but also objective DHI and quality of life scores. Due to absent vestibular data in the charts of some patients who met criteria for study inclusion, we had to exclude nearly 60 patients, which dramatically decreased the N and statistical power of our study. Despite this limitation, our study is one of the largest single-study cohorts reporting on vestibular outcomes after CI in patients with MD. While this larger cohort captured patients with both mild and more severe symptomatology, we still only analyzed 39 patients from a single institution, which does not capture the wide variability of the MD phenotype, particularly when migraine-affected patients are also included in the analysis. Thus, it may be difficult to extrapolate our results without further investigation in larger patient groups across more diverse settings.

In conclusion, this study found that in the majority of patients with MD who undergo CI in the MD-affected ear, stability or improvement of vestibular symptoms was observed. This effect appears to be mediated in patients with more severe symptomatology by the presence of comorbid migraines. Those without migraines are more likely to experience symptomatic improvement than those with migraines. Though a relatively small sample size, these findings have important implications for preoperative patient counseling, regarding the potential effects of CI on postoperative vestibular symptoms. Future studies are needed to validate these findings, using larger patient cohorts, objective vestibular symptom reports, and continued consideration of comorbid migraines.

## FUNDING SOURCES

None declared.

## CONFLICT OF INTEREST STATEMENT

S.E.M. is a paid consultant to Cochlear Americas for educational content and is a principal investigator for a sponsored trial for Cochlear Americas.

## DATA AVAILABILITY STATEMENT

Datasets generated and analyzed during the current study are available upon request to the author.
